# Patient Advocacy in Vascularized Composite Allotransplantation

**DOI:** 10.3389/fpsyg.2022.943393

**Published:** 2022-07-18

**Authors:** James Benedict

**Affiliations:** Center for Global Health Ethics, Duquesne University, Pittsburgh, PA, United States

**Keywords:** vascularized composite allotransplantation (VCA), patient advocacy, vulnerability, ethics, special protections

## Introduction

Over the last two and a half decades vascularized composite allotransplantation (VCA) has developed into a promising therapeutic option for persons who have suffered the loss of limbs, major facial disfigurement, substantial damage to the abdominal wall, primary uterine infertility and other conditions.^1^ Candidates considering VCA face a difficult choice between continuing to live with their current condition, with its substantial negative impact on their quality of life, or taking on the substantial risks and burdens of a transplant. Furthermore, the outcomes of upper extremity and facial VCA are highly dependent on the capacity and will of the patient to live with an awareness of the risks, cope with the burdens and persist in careful adherence to immunosuppression and physiotherapy. Despite certain advantages of upper extremity and facial VCA in terms of function and cosmesis, the practice has elicited a great deal of ethical concern, including concern that candidates and patients may require special protections because they are especially vulnerable (Hartman, 2007; Perpich, 2010).

This essay aims to argue that upper extremity and facial VCA candidates are rightly considered to be especially vulnerable and then explore how the provision of a patient advocate can provide protections during the screening, selection, decision-making process and post-surgical period. This argument will review the use of the concepts of vulnerability and patient advocacy in health care, describe how patient advocates were involved in some of the earliest upper limb transplantations in the United States and suggest how patient advocates in the context of upper extremity and facial VCA may be able to offset some of the concern about the vulnerability of candidates and patients.

## Vulnerability

Since its first appearance in the literature of bioethics more than 40 years ago,^2^ there has been a steadily growing interest in the concept of vulnerability. Concern for the vulnerability of research subjects and, to a lesser extent patients in clinical practice, has been expressed through the inclusion of the term in various reports, guidelines, declarations and articles.^3^ In general, the term has been used to draw attention to circumstances in which subjects or patients may be unable to adequately defend their interests.

The broadest definition of vulnerability is susceptibility to harm. This is a universal human condition, as one aspect of our natural state of mutability. Theoretical ethics has suggested our universal human vulnerability is derived from our biological and social dependence upon others.^4^ Some have argued against the relevance of a broad definition of vulnerability for bioethics (Wrigley and Dawson, 2016), but it is in fact the foundation for the well-recognized principle of non-maleficence, which is often expressed by the Latin, “*primum non nocere*,” or “first, do no harm.”

More commonly, however, bioethics is concerned with forms of special vulnerability. Special vulnerability refers to particular ways in which an individual may be more susceptible to harm than most others, due to characteristics of that individual and/or his or her context. These characteristics are thought to diminish the individual's capacity to defend against threats to his or her interests or wellbeing. An obvious example would be unconsciousness. Poverty, lack of education, disability, race and gender are also sometimes identified as sources of special vulnerability.

Vulnerability is relevant to ethics because it draws our attention to potentially avoidable or remediable human suffering. Discussions of vulnerability in health care ethics typically suggest that we have a duty to provide special protections for those who are classed as especially vulnerable (ten Have, 2014).^5^ Much of the focus on vulnerability in bioethics has been on issues involving the principle of respect for autonomy, as in cases where subjects or patients are not adequately informed, unable to process the information sufficiently, or under such duress that they are essentially coerced. Yet while limited autonomy is one source of vulnerability, there are certainly other reasons why individuals or groups may not be in a position to protect themselves. Not only may one's decisional capacity be compromised, but so too may be one's ability to carry out one's expressed preferences or desires.

In order to successfully address vulnerability in a subject, patient or population, one must first specify the vulnerability. To what exactly is the person or population vulnerable, for what reasons, and to what degree? In what ways and to what extent might the vulnerability be offset? With regard to candidates for vascularized composite allotransplantation of face or upper extremities, special vulnerability may take a number of forms.

First, they may be understood as medically vulnerable. Medical vulnerability applies to candidates for VCA because it refers to persons who are so seriously ill or injured that they may be attracted to research protocols by unrealistic expectations (Benvenuti et al., 2021). As Nickel points out, “In general, research subjects tend to underestimate the level of risk or impact associated with participation in biomedical research,” while overestimating the likelihood of potential benefits. Subjects do so, Nickel claims, because of the effects of “'motivated bias,' i.e., errors based on a desire to believe something is true.” (Nickel, 2006) Given that those drawn to VCA tend to be those who have been unable to adapt to their disfigurement or disability, while others of equal or greater injury do adapt, the question is raised whether VCA inadvertently targets the most desperate among the disabled and disfigured who may therefore also be the most vulnerable (Rumsey, 2004; Bradbury, 2012).

Second, candidates for VCA may be understood as socially vulnerable. Social vulnerability applies to candidates for VCA whose condition has led to social isolation. Facial defects often lead to such isolation (Strandmark, 2004; Strong, 2004; Svenaeus, 2012).^6^ Upper extremity defects may also cause isolation, as a result of their effect on the individual's self-image or because the functional consequences of the defect exclude the individual from certain activities. Examples would be the loss of the ability to continue one's career, certain activities of daily living or familiar leisure activities. A sense of social isolation may also be created by the increased level of dependency that occurs as a result of a defect.

Loss of independence due to disability has been associated with a decrease in psychological wellbeing and subjective estimates of quality of life, a limitation of employment opportunities, and social stigma or marginalization. Persons with disabilities frequently “report giving up established ways of doing things, and forgoing numerous activities, plans and goals.” Various factors have been identified as playing a role in the subjective perception of dependence, including not only an individual's pre-existing coping skills, but the “cultural norms and societal values” to which the individual has been exposed (Gignac and Cott, 1998). When facial defects are of such a nature as to prevent normal eating or even normal breathing, and when upper extremity defects render persons unable to drive, maintain employment, feed themselves, etc., a state of dependency may be created which individuals may be so anxious to escape that they are willing to take far greater than normal risks.

Third, candidates for VCA may be deemed vulnerable due to the complexity of the decision and the limits of imagination (Fischer et al., 2021). In order to make a decision, candidates must attempt to imagine a future in which they will be confronted with major burdens and risks. While they may receive substantial benefits, they are also taking on what amounts to a kind of chronic illness, some aspects of which will diminish their quality of life. They may struggle to cope with the side effects of immunosuppression or the rigors of physiotherapy.^7^ Upper extremity recipients will endure a period of time, often several months, during which they will actually be less functional and more dependent than before the surgery. They must try to imagine how the treatment and the transformation thus wrought will affect their relationships with family members, friends, or co-workers. Additionally, all candidates should assume that a time will come when the graft will be lost. In order to make a sound decision to proceed, candidates must imagine life under these conditions and determine whether or not they will be able to rise to the occasion.^8^

In addition to these forms of special vulnerability which apply to VCA candidates and may compromise their ability to provide adequate initial consent, it should be recognized that the treatment itself imposes upon the recipient new forms of vulnerability which must be borne by the patient thereafter. A recipient is susceptible to harm from comorbidities associated with the surgery, post-surgical infections and both acute and chronic graft rejection. The recipient is also susceptible to harms associated with the long-term use of immunosuppression, such as increased infection risk, the development of diabetes, kidney damage, and increased rates of malignancy (O'Neill and Godden, 2009; Hautz et al., 2011; Shores et al., 2011; Pomahac et al., 2012). In order to manage this new vulnerability, the recipient must carefully maintain the schedule of immunosuppression, participate actively in physiotherapy for years, and self-monitor for signs of rejection for the rest of his or her life. The patient must also reckon with the likelihood that the decision he or she has made to have the transplant may contribute directly to an earlier death. Support in coping with the complications and carrying out the responsibilities is one means of offsetting the added vulnerability.

## Patient Advocacy

The terms “patient advocacy” and “patient advocate” appear frequently in the literature of healthcare but lack any singular or settled definition. “Patient advocacy” has been used to describe efforts of patients themselves to obtain access to or improve treatment (Epstein, 1995; Brashers et al., 2000), as a description of a central feature of the nursing ethos (Bu and Jezewski, 2007; Choi, 2015), and as a description of individuals whose primary role is to assist patients in navigating their way through the complexities of modern health care systems.^9^ Patient advocacy in all its forms exists to redress conditions which place patients at a disadvantage, particularly power differentials between patients and providers or patients and systems (Erlen, 2006; Reid, 2022).

Patient advocacy in the form of assisting patients as they navigate their way through treatment may assume different foci at different times. It may focus on pursuit of the patient's best interests, protection and promotion of the patient's rights, formal representation of the patient, or empowerment of the patient by providing information, assuring understanding and providing emotional support (Brazg et al., 2016; Abbasinia et al., 2020). Regardless of the particular focus, the primary duty of the patient advocate is to the vulnerable patient. In the words of Bragz et al., “Patient advocacy is one response to patients' experiences of vulnerability, and it can be utilized as a tool to improve patients' participation and engagement in their healthcare.”^10^

The value of a patient advocate has already been recognized in research (Cauhan and Eppard, 2004; Katz et al., 2012; Salamone et al., 2018) and in the field of transplantation. In the context of living donation, the Organ Procurement and Transplantation Network (OPTN) requires the involvement of an independent living donor advocate (ILDA). The responsibilities of this particular kind of patient advocate are to “represent, advocate, protect and promote the best interests” of those who express an interest in donating an organ while alive, by providing information about the process and risks, ensuring free, uncoerced and fully informed consent and providing support for those prospective donors who are not allowed to donate.^11^ The assumptions behind the requirement for an ILDA is that potential donors may be vulnerable due to a lack of knowledge or failure to appreciate the burdens, risks and possible negative outcomes for both donor and recipient. Potential donors may also be vulnerable to coercion, especially if the person in need of a transplant is a spouse, sibling, parent or child of the donor.

## The Louisville Experience

From 1999^12^ to 2011, a team in Louisville, Kentucky, involving the Jewish Hospital, the Christine M. Kleinert Institute for Hand and Microsurgery and the University of Louisville, performed hand transplantation on six patients. Preparation for these transplants began in 1995 when a group of hand surgeons, transplant surgeons, psychiatrists, nephrologists, physical therapists, nurses, tissue typing lab specialists, ethicists and organ procurement organization representatives came together to envision how to create a program. Initial discussions led to a commitment to undertaking a great deal of basic science research and work in large animal (swine) models prior to attempting a transplant on a human patient.

Another major commitment of the Louisville program from the very start was a commitment to ethical reflection, transparency and accountability. The program sought out advice from Dr. Siegler, director of MacLean Center for Clinical Medical Ethics at the University of Chicago. Among Siegler's recommendations was that the team should announce its intentions prior to its first attempt, rather than wait to see whether the procedure would be a success before deciding if it would be publicized. This approach would heighten their accountability. Another recommendation from Dr. Siegler was that the team consider including a patient advocate for each patient. This recommendation was embraced, and a patient advocate was involved for each of the first six patients at Louisville.

The first meeting with a prospective patient began with introductions to the surgeon and staff. The patient was asked to explain why they wanted a hand transplant, and given an initial introduction to the risks. Psychological and general medical evaluation followed, and the first encounter ended with the candidate being urged to consider the options and risks thoroughly before deciding whether to schedule of second appointment.

At the second appointment, a much more detailed presentation of the procedure and potential complications took place. A potential patient was also informed that a 6-month trial with a prosthesis would be mandatory before the patient could become eligible for a transplant. The patient was also informed of the need for further psychological testing and that an analysis of the family and social support system would have to take place.

After 6 months or more, if the outcomes of the various screenings were acceptable and the patient continued to be interested, the patient was introduced to the concept of a patient advocate. It was explained that the patient was expected to select his or her own advocate, who should be someone who knew them well but not a family member. The role of this advocate was to accompany the patient through the remaining process prior to surgery and help the patient reach a free and informed decision. The ideal patient advocate was someone who had at least some familiarity with medical terms, the ability to identify and weigh burdens, risks and potential benefits, and the ability to construct and communicate a recommendation.

The advocate would have access to all the information about the treatment that was available to the patient, and could ask questions of the treatment team. The transplant team was prohibited from trying to exert influence over the advocate in any way. While the advocate would make a recommendation on whether to proceed, the final decision was up to the patient.

## Patient Advocacy for VCA Candidates and Recipients

If it is accepted that VCA candidates and recipients are properly regarded as being especially vulnerable, and that patient advocacy is a reasonable way of addressing the needs of vulnerable patients in health care, then the provision of patient advocates for VCA candidates and recipients may be recognized at least as a moral good, and perhaps as an ethical duty. The anticipated role of a patient advocate in VCA would include (but not necessarily be limited to) (1) assistance in the pursuit of information and in the deliberation leading up to the decision of whether or not to give formal consent and (2) continuing support through rehabilitation and adjustment to post-transplant life (Caplan et al., 2019).

Important traits and skills for patient advocacy in VCA include independence from both the health care team and from the patient. Ideally, the patient advocate should be neither an employee of the health care system providing the treatment nor a family member or intimate friend of the patient. Advocates should know the patient well-enough to have a sense of their values and to be aware of their psychosocial strengths and weaknesses, yet not be so close that the advocate would hesitate to challenge the patient's thinking. Independence from both the team and the patient allows the advocate to express himself or herself without excessive concern about how it may be received by either the care team or the patient. Some degree of health care literacy would be important, so that the advocate would not have difficulty understanding information and could potentially “translate” information for the patient as needed. Communication skills are obviously essential, as is psychosocial stability and emotional intelligence.^13^

The involvement of a patient advocate in VCA does not imply that candidates or patients need protection from the transplantation team, *per se*. Rather, it is based on the recognition that even the most caring and careful transplantation team is inherently limited in its ability to address the full range of the patient's vulnerability. Despite best intentions and efforts, it may be impossible for the team to adequately appreciate the strengths, weaknesses and perspectives of the patient and his or her support system.^14^ Evidence of the difficulty of doing so is reflected in the frequent assertion that improvement in patient selection is a primary need in the field (Edwards and Mathes, 2011; Kiwanuka et al., 2013; Jowsey-Gregoire et al., 2016; Shores et al., 2017). It may also be impossible for the team to adequately address the post-surgical vulnerability of the patient, particularly when he or she lives at some distance from the center where the team practices.

Likewise, the involvement of a patient advocate does not imply that the patient lacks decisional capacity. The patient advocate does not serve as a surrogate decision maker, but as a trusted counselor. The patient advocate may raise questions, help assure patient comprehension, and offer opinions, but the patient advocate should not be given the authority to override the patient's choice.

The involvement of an independent patient advocate also provides ethical protection for both patients/candidates and transplantation teams who share in the universal predisposition to self-justification and self-deception. The investments of time and money that must be made to establish VCA programs, as well as the potential rewards in terms of the economy of fame,^15^ create significant pressures on individuals to justify what they desire or what is necessary for them to achieve a given status. This results in the creation of “confirmation bias” (Haynes and Haynes, 2009; Mendel et al., 2011) in the assessment of candidates. The same pressures may apply to candidates, who may engage in similar practices of self-justification and self-deception. The role of the patient advocate is to be an independent interlocutor, who can raise questions, challenge reasoning, and offer alternative perspectives.

## Conclusion

Candidates for upper extremity and facial VCA exhibit characteristics associated with special social and medical vulnerability. In addition, the complexity and relative lack of data on these forms of VCA increase their vulnerability. It is an established practice elsewhere in some research and in living donor transplantation to provide a patient advocate to support, advocate for and protect subjects and patients. Furthermore, the use of patient advocates early in the Louisville program demonstrates the feasibility of incorporating patients advocates into VCA practice. It is therefore recommended that serious consideration be given to the recruitment, training and use of patient advocates in upper extremity and facial VCA in the future.

## Author Contributions

The author confirms being the sole contributor of this work and has approved it for publication.

## Conflict of Interest

The author declares that the research was conducted in the absence of any commercial or financial relationships that could be construed as a potential conflict of interest.

## Publisher's Note

All claims expressed in this article are solely those of the authors and do not necessarily represent those of their affiliated organizations, or those of the publisher, the editors and the reviewers. Any product that may be evaluated in this article, or claim that may be made by its manufacturer, is not guaranteed or endorsed by the publisher.

## Footnotes

^1^Other types of VCA that have been attempted or seriously considered include transplantation of the esophagus, larynx, knee, penis, and tongue.

^2^For a detailed accounting of the use of the term in bioethics, see ten Have (2016).

^3^Examples include the Belmont Report, the Council for International Organizations of Medical Sciences (CIOMS) Guidelines. The UNESCO Universal Declaration on Bioethics and Human Rights, and the Declaration of Helsinki. Recent articles treating the topic include Monacelli et al. (2016), Amgel and Vatne (2017), and Bracken-Roche et al. (2017).

^4^See Goodin (1985), Nussbaum (1986), and MacKenzie et al. (2014). This idea of dependence as a natural state is traditional, going back in the western tradition at least to the divine declaration in Genesis 2:18. “It is not good for the man to be alone.”

^5^The Principle of Respect for Human Vulnerability and Personal Integrity: Report of the International Bioethics Committee. Available online at: http://unesdoc.unesco.org/images/0021/002194/219494E.pdf (accessed January 25, 2014)

^6^Rumsey (2004, p. 22–23) and Bradbury (2012, p. 193–196).

^7^Failures to cope with their post-transplant condition have been the source of complications, including graft loss in some cases. See Tintle et al. (2014) and Kumnig and Jowsey-Gregoire (2016).

^8^May captures the profound challenge of illness and treatment for patients. He perceptively reminds us. “[T]o equate the heroic with the aggressiveness of the doctor's technical interventions cruelly overlooks those who bear the true weight of heroism.... the heavy burden of heroism in medicine falls not on the physician but on the patient and the patient's family, as they often face, after the successful rescue, an extraordinarily long and heavy responsibility of chronic care.” (William, 1991).

^9^See, for example, the website of the Alliance of Professional Health Advocates. Available online at: https://www.aphadvocates.org/profession-overview/ (accessed September 9, 2017).

^10^Bragz, et al., 177. See also Erlen, 134, who contends that advocacy “may reduce the level of vulnerability.”

^11^OPTN has also extended the requirement for an ILDA to VCA, in the event of living donation. At present, uterine transplantation may use living donors; other possibilities for living donation in VCA have been imagined as well. See “VCAs from Living Donors. US Department of Health and Human Services OPTN (Vittone and Crowell, 2021). Available online at https://optn.transplant.hrsa.gov/resources/by-organ/vascular-composite-allograft/vcas-from-living-donors/ (accessed September 9, 2017) (Hays et al., 2015; Vittone and Crowell, 2021).

^12^Information about the Louisville program was obtained through personal communication with Warren Breidenbach, MD, who led the program at Louisville from 1999-2011.

^13^Emotional intelligence is defined as “the ability to monitor one's own and other people's emotions, to discriminate between different emotions and label them appropriately, and to use emotional information to guide thinking and behavior.” See Oxford University Press (2015).

^14^At least one such program goes so far as to spend several days visiting in the candidate's home community, attempting to gain a better understanding. (Personal communication with Eduardo Rodriguez, of NYU-Langone Medical Center).

^15^For an excellent article on the role of the economy of fame in the development of science, see Franck (2015).
